# Wind‐energy development alters pronghorn migration at multiple scales

**DOI:** 10.1002/ece3.9687

**Published:** 2023-01-10

**Authors:** Megan C. Milligan, Aaron N. Johnston, Jeffrey L. Beck, Kaitlyn L. Taylor, Embere Hall, Lee Knox, Teal Cufaude, Cody Wallace, Geneva Chong, Matthew J. Kauffman

**Affiliations:** ^1^ U.S. Geological Survey Northern Rocky Mountain Science Center Bozeman Montana USA; ^2^ Department of Ecosystem Science and Management University of Wyoming Laramie Wyoming USA; ^3^ Grouse Mountain Environmental Consultants Buffalo Wyoming USA; ^4^ Wyoming Game and Fish Department Laramie Wyoming USA; ^5^ Wyoming Cooperative Fish and Wildlife Research Unit, Department of Zoology and Physiology University of Wyoming Laramie Wyoming USA; ^6^ U.S. Geological Survey, Wyoming Cooperative Fish and Wildlife Research Unit, Department of Zoology and Physiology University of Wyoming Laramie Wyoming USA

**Keywords:** *Antilocapra americana*, energy development, fidelity, migration, pronghorn, wind‐energy development

## Abstract

Migration is a critical behavioral strategy necessary for population persistence and ecosystem functioning, but migration routes have been increasingly disrupted by anthropogenic activities, including energy development. Wind energy is the world's fastest growing source of electricity and represents an important alternative to hydrocarbon extraction, but its effects on migratory species beyond birds and bats are not well understood. We evaluated the effects of wind‐energy development on pronghorn migration, including behavior and habitat selection, to assess potential effects on connectivity and other functional benefits including stopovers. We monitored GPS‐collared female pronghorn from 2010 to 2012 and 2018 to 2020 in south‐central Wyoming, USA, an area with multiple wind‐energy facilities in various stages of development and operation. Across all time periods, we collected 286 migration sequences from 117 individuals, including 121 spring migrations, 123 fall migrations, and 42 facultative winter migrations. While individuals continued to migrate through wind‐energy facilities, pronghorn made important behavioral adjustments relative to turbines during migration. These included avoiding turbines when selecting stopover sites in spring and winter, selecting areas farther from turbines at a small scale in spring and winter, moving more quickly near turbines in spring (although pronghorn moved more slowly near turbines in the fall), and reducing fidelity to migration routes relative to wind turbines under construction in both spring and fall. For example, an increase in distance to turbine from 0 to 1 km translated to a 33% and 300% increase in the relative probability of selection for stopover sites in spring and winter, respectively. The behavioral adjustments pronghorn made relative to wind turbines could reduce the functional benefits of their migration, such as foraging success or the availability of specific routes, over the long term.

## Graphical abstract

Wind energy is the world's fastest growing source of electricity and represents an important alternative to hydrocarbon extraction, but its effects on migratory species beyond birds and bats are not well understood. We found that while connectivity was not lost and pronghorn continued to migrate through wind‐energy facilities, pronghorn made important behavioral adjustments relative to turbines during migration. These included avoiding turbines when selecting stopover sites, selecting areas farther from turbines at a small scale, moving more quickly near turbines in the spring, and reducing fidelity to migration routes relative to wind turbines under construction.

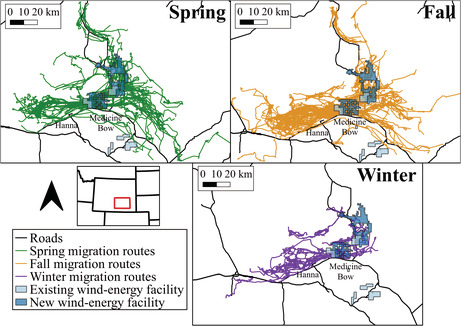

## 1 INTRODUCTION

Migration is an impressive and critical behavioral strategy that allows ungulates to access seasonal resources, avoid severe weather conditions, and track gradients in high‐quality forage (Fryxell & Sinclair, 1988; Harris et al., 2009; Sawyer et al., 2009). Migratory species can be important drivers of ecosystem processes such as maintaining biodiversity, but the widespread decline of ungulate migrations highlights the need for effective conservation (Bolger et al., 2008; Harris et al., 2009; Runge et al., 2014). Ungulate migrations have been significantly disrupted by anthropogenic activities, and features such as roads, fences, and pipelines increasingly intersect migration routes, disrupting or impeding movement (Bolger et al., 2008; Harris et al., 2009; Sawyer et al., 2013). Such barriers can eliminate migratory populations entirely or increase rates of residency, which can have significant population‐level consequences including local extirpations (Bolger et al., 2008; Harris et al., 2009; Mueller et al., 2011). Given the important ecosystem functions of migratory herbivores, such as influencing biodiversity and species distributions and the role of migration in allowing individuals to persist by accessing seasonally important resources, conserving both migration corridors and migratory species has been recognized as a conservation priority (Kauffman et al., 2021).

Global energy extraction has increased in recent decades and is responsible for altering migration routes throughout the world (Leu et al., 2008; Sawyer et al., 2009). For example, anthropogenic development led to a decline in the number of migratory shorebirds using the East Asian‐Australasian Flyway (Murray et al., 2014) and disrupted ungulate migrations in Asia and Africa (Berry, 1997; Nandintsetseg et al., 2019). Oil and gas development has disrupted ungulate migrations in the western United States, with the impacts to mule deer (*Odocoileus hemionus*) migrations being the best understood. While routes were often maintained (Lendrum et al., 2012; Wyckoff et al., 2018), deer increased speed when migrating through areas of high development and reduced time in stopover sites (Lendrum et al., 2012; Sawyer et al., 2013; Wyckoff et al., 2018), a key habitat where animals linger during migration to forage and keep pace with the wave of spring green‐up (Sawyer et al., 2009; Sawyer & Kauffman, 2011). The intensity of use and the total area of migration routes has declined sharply even at low levels of surface disturbance (Sawyer et al., 2013, 2020). Changes in fidelity can result in the total loss of migration routes used by a population, whereas shifts in stopover sites and small‐scale avoidance can translate to functional habitat loss, both of which can have potential population‐level consequences (Sawyer et al., 2017). Such displacement can restrict the amount of habitat available and reduce access to important forage resources, thus reducing fitness and population viability, particularly in populations with spatially restricted habitat and where habituation does not occur (Aikens et al., 2022; Sawyer et al., 2017). Despite numerous studies documenting the influence of oil and gas development on mule deer migration, we know little about the impacts of alternative forms of energy development on other ungulate species. Wind‐energy development is the world's fastest growing source of electricity (Jones & Pejchar, 2013), driven by goals to reduce carbon emissions (Allison et al., 2019). While the effects of wind‐energy development on bird and bat migrations have been well‐studied (Allison et al., 2019), there is little information on its effects on ungulate migration and whether they are similar to the effects documented for oil and gas development. Given that ungulates may respond differently to disturbances unique to wind energy (Jones & Pejchar, 2013), and that migration is a critical life‐history trait for many populations, understanding how different forms of development, including wind energy, impact migratory behavior is important for management and conservation.

Pronghorn (*Antilocapra americana*) are a culturally and economically important ungulate species endemic to western North America (O'Gara & Yoakum, 2004). Energy development has increased throughout the species' range, including multiple existing and proposed wind‐energy developments in the critical winter range (WGFD, 2011). Previous research suggested that wind‐energy development had highly variable effects on habitat selection in both summer and winter (Milligan et al., 2021; Smith et al., 2020). Prior research has documented the prevalence of migratory behavior in pronghorn (Jacques et al., 2009; Jakes et al., 2018; Kolar et al., 2011; Tack et al., 2019), but no study has evaluated the effects of wind‐energy development on migratory behavior for this species. Pronghorn are conditionally migratory, with >50% of individuals switching movement strategies between years and populations using facultative winter migrations to mitigate harsh weather conditions (Jacques et al., 2009; Jakes et al., 2018; Larkins et al., 2018; Tack et al., 2019). Species that exhibit high fidelity to narrow pathways, such as moose (*Alces alces*) and mule deer (Morrison et al., 2021; Sawyer et al., 2009; Sawyer & Kauffman, 2011; Wyckoff et al., 2018), may be more vulnerable to disruptions to migration routes than populations that are more nomadic or flexible such as Mongolian gazelle (*Procapra gutturosa*; Mueller et al., 2011) and pronghorn. Migration is nevertheless critical to the survival and persistence of semi‐nomadic species, particularly when migrations allow individuals to respond to severe weather conditions as documented for pronghorn (Jakes et al., 2018; Tack et al., 2019).

Our objective was to evaluate the effects of wind‐energy development on pronghorn migration, including behavior and habitat selection. We sought to answer five questions: (1) do pronghorn migrate through wind‐energy facilities, (2) does the behavior of pronghorn that move through wind‐energy facilities differ from those that do not, (3) does fidelity to either routes or stopover sites differ relative to wind turbines, (4) does proximity to wind turbines influence the selection of migration routes or stopover sites at a landscape scale, and (5) do migrating pronghorn alter habitat selection or movement behavior at a small scale in proximity to turbines? We predicted that pronghorn would still migrate through wind‐energy facilities and not alter their route selection based on turbines, but that animals would alter their behavior, potentially migrating at faster speeds near turbines and selecting habitats at a small spatial scale to avoid turbines.

## 2 STUDY AREA

**FIGURE 1:**
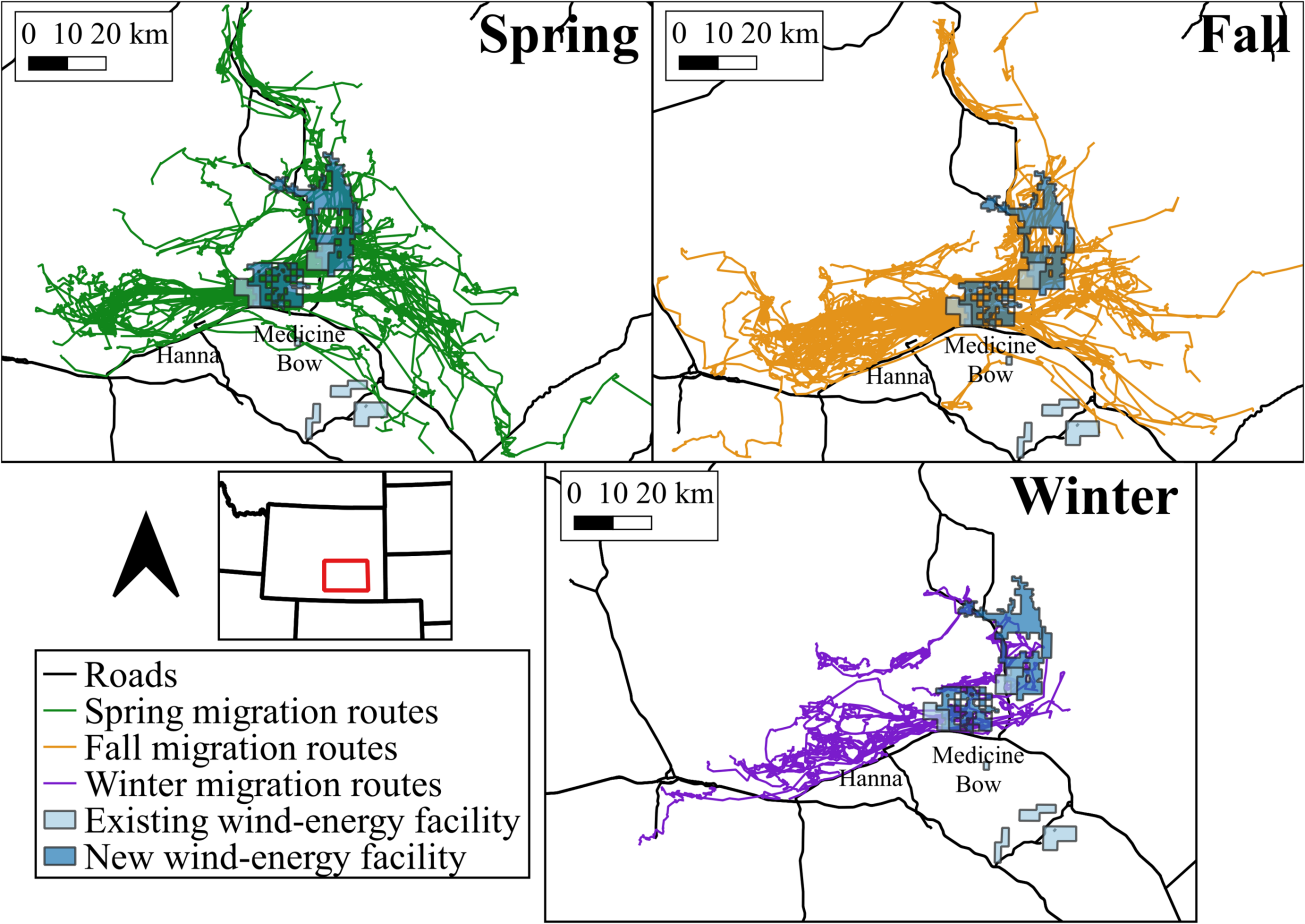
Study area in south‐central Wyoming, USA, with individual spring (green), fall (orange), and facultative winter (purple) migration routes, 2010–2012 and 2018–2020. Each line is the path of an individual migration route. The footprints of existing wind‐energy facilities (light blue) and new wind‐energy facilities where construction began in summer 2019 (dark blue) are shown.

We monitored pronghorn in areas with wind‐energy facilities near Medicine Bow in Carbon and Albany Counties, Wyoming, USA (Figure 1). The area is dominated by arid shrublands and grasslands, with Wyoming big sagebrush (*Artemisia tridentata wyomingensis*) as the most prevalent cover type. Elevations ranged from 1320 to 3350 m. Average temperatures (Western Regional Climate Center, 2021) ranged from −8.76 to 19.93°C in spring (March–May), −12.75 to 24.29°C in fall (Sept–Nov), and −19.86 to −2.13°C in winter (Dec–Feb). Our study area was centered around two existing wind‐energy facilities and two facilities under construction. The Seven Mile Hill wind‐energy project had 79 turbines that became operational in December 2008. The Dunlap Ranch wind‐energy facility had 74 turbines that were constructed from September 2009 to September 2010. Construction of Ekola Flats (63 turbines) and TB Flats (132 turbines) wind facilities began in April 2019 and was completed by July 2021. Construction activity ceased in late December due to winter weather and restarted in March. Four additional wind‐energy facilities were operational prior to pronghorn tracking and were located on the periphery of the study area: Little Medicine Bow (10 turbines), Foote Creek Rim (100 turbines), Rock River (50 turbines), and High Plains (85 turbines).

## 3 METHODS

### 3.1 Pronghorn capture and tracking

Pronghorn were monitored with store‐on‐board GPS collars (model G2110B; Advanced Telemetry System, Isanti, MN and RECON‐4560‐4; Telonics, Mesa, AZ) from 2010 to 2012 and 2018 to 2020. Thirty‐five and 17 adult female pronghorn were initially captured using helicopter net gunning (Leading Edge Aviation, Lewiston, ID, USA) in January 2010 and December 2011, respectively, and monitored through May 2012. Eighty adult female pronghorn were captured in March 2018 (Native Range Capture Services, Elko, NV, USA), with additional captures of 20, 13, 16, and 37 females in December 2018, March 2019, November 2019, and March 2020, respectively, and monitored through summer 2020. Capture and handling protocols were approved by the University of Wyoming Institutional Animal Care and Use Committee (protocols 01012010 and 20180306MK00297‐03) and Wyoming Game and Fish Department (Chapter 33 Permit IDs 742 and 1162). During 2010–2012, fix rates were 11 h from 16 May to 15 November and 7 h from 16 November to 15 March. We recovered these collars by May 2012. During 2018–2020, collars had 2‐h fix rates and transmitted every fourth location via satellite. We obtained the 2‐h data from 90 individuals that died during the second study period.

### 3.2 Defining migration routes and stopover sites

We classified individuals as resident, migratory, or mixed migratory (i.e., using multiple ranges or not migrating every season) and identified migration start and end dates using net‐squared displacement (Bunnefeld et al., 2011). To identify spring, fall, and facultative winter migrations, we then calculated utilization distributions (30‐m resolution) for each route using Brownian bridge movement models (Horne et al., 2007) from the “BBMM” package in Program R (Nielson et al., 2013; R Core Team, 2019). Facultative winter migrations were defined for individuals that migrated after 1 January or subsequent direct movements from an initial winter range to a spatially distinct alternative winter range and constrained to start after an individual was in the winter range for >4 weeks. For individuals with 8‐h fix rates (*n* = 128), we set the motion variance equal to the median of the motion variances calculated for individuals with 2‐h data (median = 3000). No pronghorn migrated along the same path at the same time, so we considered all individuals independent. We classified stopover sites for individuals based on the top 10% area contour of the utilization distribution from the Brownian Bridge movement models (Sawyer et al., 2009).

### 3.3 Habitat variables

We identified 10 habitat factors a priori that could influence the selection of migration routes: sagebrush (*Artemisia* spp.) and herbaceous cover (Xian et al., 2015), terrain ruggedness (Riley et al., 1999), vegetation quality, vegetation phenology in spring, snow depth (1 km^2^ daily resolution; National Operational Hydrologic Remote Sensing Center, 2004), roads, fences, and wind turbines (Johnston et al., 2022). For vegetation quality, we used integrated normalized difference vegetation index (iNDVI) from the Moderate Resolution Imaging Spectroradiometer (MODIS; Johnston et al., 2018). For phenology, we calculated the instantaneous rate of green‐up in spring using a fitted curve to the annual NDVI time series from MODIS (Bischof et al., 2012). We digitized fences based on aerial imagery from the National Agricultural Imagery Program.

### 3.4 Data analysis

#### 3.4.1 Movement behavior

We quantified the number and proportion of routes that traveled through wind‐energy facilities in each season and then compared metrics of movement behavior relative to the proportion of the total route within 1 km of turbines. For each route, we calculated general metrics, including the Euclidean and total path length, average speed (km/h) over each path length, and total duration of migration. We used linear regression in a Bayesian framework with random intercepts for individual pronghorn implemented in the “rstanarm” package in Program R and the Watanabe‐Akaike Information Criterion (WAIC; Hooten & Hobbs, 2015) to compare models containing an effect of the proportion of a route within 1 km of turbines, season, and an interaction between the two variables.

In addition, we identified important habitat factors influencing migration speed in each season and then evaluated those factors in combination with distance to turbine for steps within 20 km of turbines. We calculated speed (km/h) for all steps between consecutive locations of a migration route and extracted habitat variables at the starting location of each step. We log‐transformed speed and modeled distance to turbine as a linear effect because log‐transformation did not improve fit and evaluated models as described above. We also compared movement rates from before to during the construction of Ekola Flats and TB Flats using an unpaired two‐sample Wilcoxon test (*α* < 0.05) for individuals that migrated within 1 km of turbine sites.

#### 3.4.2 Fidelity

We evaluated fidelity to migration routes and stopover sites for individuals tracked for two years (spring migrations *n* = 25, fall *n* = 32) by comparing the overlap between successive routes and stopover sites. Overlap was calculated as the proportion of an individual's migration route or stopover from the second monitoring year that overlapped the same individual's route in the first monitoring year. For individuals monitored from 2018 to 2020, we evaluated whether the proximity of an individual's route to turbines under construction influenced fidelity to spring (*n* = 21) and fall (*n* = 30) migration routes and stopover sites. We used linear regression in a Bayesian framework with a beta distribution and used WAIC to evaluate models with an effect of season, proximity to turbine, and an interaction between season and proximity to turbine on the proportion overlap of either migration routes or stopover sites, basing inferences on the model with the lowest WAIC.

#### 3.4.3 Migration route and stopover selection

We used conditional logistic regression in a Bayesian framework in a matched use‐available design to compare used migration routes to available routes with the same start and end points (Manly et al., 2002). We defined used migration routes using all locations in which an animal was moving forward with a turning angle ≤|90°| to exclude stopover sites. We then generated available routes of the same length and duration with the same start and end points at a 10:1 available:used ratio and weighted available routes (*w* = 1000) to improve coefficient estimates and model convergence (Northrup et al., 2013). We calculated the average of each habitat covariate along a migration route. For time‐varying covariates (e.g., snow depth), we calculated an average value for each route during migration. We used the minimum distance to existing turbines to evaluate the effects of wind facilities. We log‐transformed all distance measures to allow the effect to decline at farther distances and scaled and centered all fixed effects. We did not include herbaceous cover and integrated NDVI in the same model because of high correlation (*r* ≥ 0.6) in both spring and fall. We used univariate models and WAIC to identify important habitat variables to combine with roads and fences. We then added turbine variables to the top model retained from the previous step. We based inferences on the model with the lowest WAIC and retained parameters whose 95% credible intervals did not overlap zero. We used the R‐INLA package to fit models with stratum‐specific intercepts for matched used and available routes modeled as random effects with large fixed variance *α* ~ *N*(0, 10^6^). We considered coefficients with 95% credible intervals that did not overlap zero to be significant.

We evaluated the effects of turbines under construction on route selection following the same procedures described above in separate models because of high correlation with distance to existing turbines. We only included individuals that migrated during the construction period and did not evaluate winter migration due to small sample sizes. We excluded sagebrush cover for spring migration routes and snow depth for fall migration routes because of high correlation with distance to turbine under construction.

We used the same analytical approach to compare attributes of stopover polygons to available polygons selected randomly from within each individual's route at a 10:1 available:used ratio. We calculated habitat variables within each polygon and measured the average distance of polygons to turbines. We also evaluated the amount of time individuals spent in stopover sites, using the number of points within a stopover polygon as a proxy for time and relating that to distance to turbine using linear regression.

#### 3.4.4 Small‐scale selection

We used integrated step‐selection functions with random slopes for individuals (Muff et al., 2019) to evaluate the effect of wind turbines on fine‐scale habitat selection and movement behavior (Avgar et al., 2016). We used the “amt” package in Program R to format the location data into steps and randomly sample 3 available steps for every used step (Signer et al., 2019). Step lengths and turning angles for available steps were drawn from a Gamma and a von Mise's distribution, respectively (Avgar et al., 2016; Signer et al., 2019). All models included log‐transformed step length (log[step length]) and the cosine of the turning angle (cos[turn angle]) to account for directional persistence, allowing for unbiased inferences regarding habitat selection and movement (Avgar et al., 2016). We evaluated the habitat variables defined above at the end of a step by comparing all single‐variable models using WAIC. All variables that improved model fit over the null model were included in the base model, which we evaluated with all possible combinations of fence and road variables. The top model provided the foundation for the three models that tested for the effects of wind turbines on pronghorn migration behavior. The first model included distance to wind turbine at the end of a step to evaluate whether pronghorn selected habitat along the migration route relative to turbines. The second model included distance to turbine in interaction with log(step length) to evaluate whether pronghorn changed speed with proximity to wind turbines (Avgar et al., 2016). The third model included distance to turbine in an interaction with cos(turn angle) to evaluate whether pronghorn changed direction in proximity to turbines, potentially detouring around turbines. We also evaluated turbine effects separately from habitat factors to evaluate the relative importance of each group of variables. We considered coefficients with 95% credible intervals that did not overlap zero to be significant. Sample sizes were inadequate to evaluate the effects of turbines under construction on small‐scale selection. We used the R‐INLA package (Rue et al., 2009) to fit conditional Poisson models with random slopes with stratum‐specific intercepts for matched used and available steps modeled as random effects with large fixed variance *α* ~ *N*(0, 10^6^) and penalized complexity priors, PC(3, 0.05), for the precision of random slopes for all habitat variables.

## 4 RESULTS

Overall, we classified 101 pronghorn as residents (*n* = 60) or as not having sufficient data to be classified due to short monitoring periods (*n* = 41), 43 were classified as migratory, and 74 were mixed migratory. We collected 286 migration sequences from 117 individuals (Figure 1), including 121 spring migrations, 123 fall migrations, and 42 facultative winter migrations. Of the 286 migrations, 200 (70%) passed within 1 km of a wind turbine and 226 (79%) passed within 5 km of a wind turbine. Both the metrics and timing of migrations varied by both season and year (Table S1).

### 4.1 Movement behavior

**FIGURE 2:**
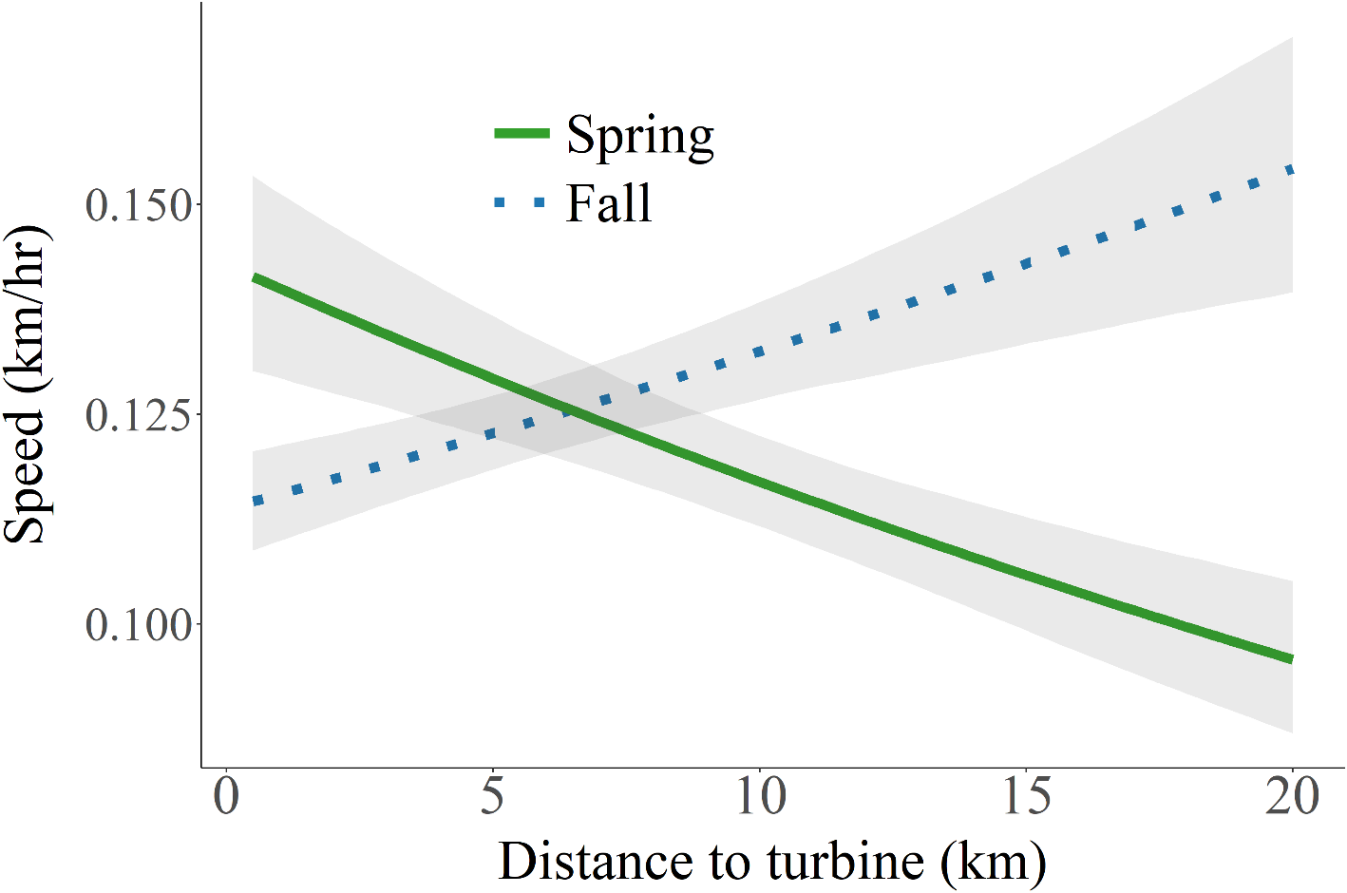
Predicted speed (km/h) of migrating female pronghorn relative to turbines with other habitat variables (spring: instantaneous rate of green‐up; fall: sagebrush cover) held at mean values.

General migration metrics, including the Euclidean and total path length, average speed (km/h) over each path length, and total duration of migration, differed among seasons, but not relative to the proportion of a route near turbines (Table S2). The instantaneous rate of green‐up and sagebrush cover were the most important habitat factors influencing speed in spring and fall, respectively, but distance to turbine was still an important predictor even after controlling for other habitat factors (Figure 2, Table S3). When near turbines, pronghorn moved more slowly in fall but more quickly during spring (Figure 2). Neither habitat nor turbine variables were strong predictors of speed during winter. There was no difference in movement rates between routes that traveled through either Ekola Flats or TB Flats prior to construction compared with routes in the same area during construction (preconstruction: 0.29 ± 0.03 km/h, during construction: 0.36 ± 0.07, *p* = .49), although sample sizes prevented us from evaluating differences across seasons.

### 4.2 Fidelity

**FIGURE 3:**
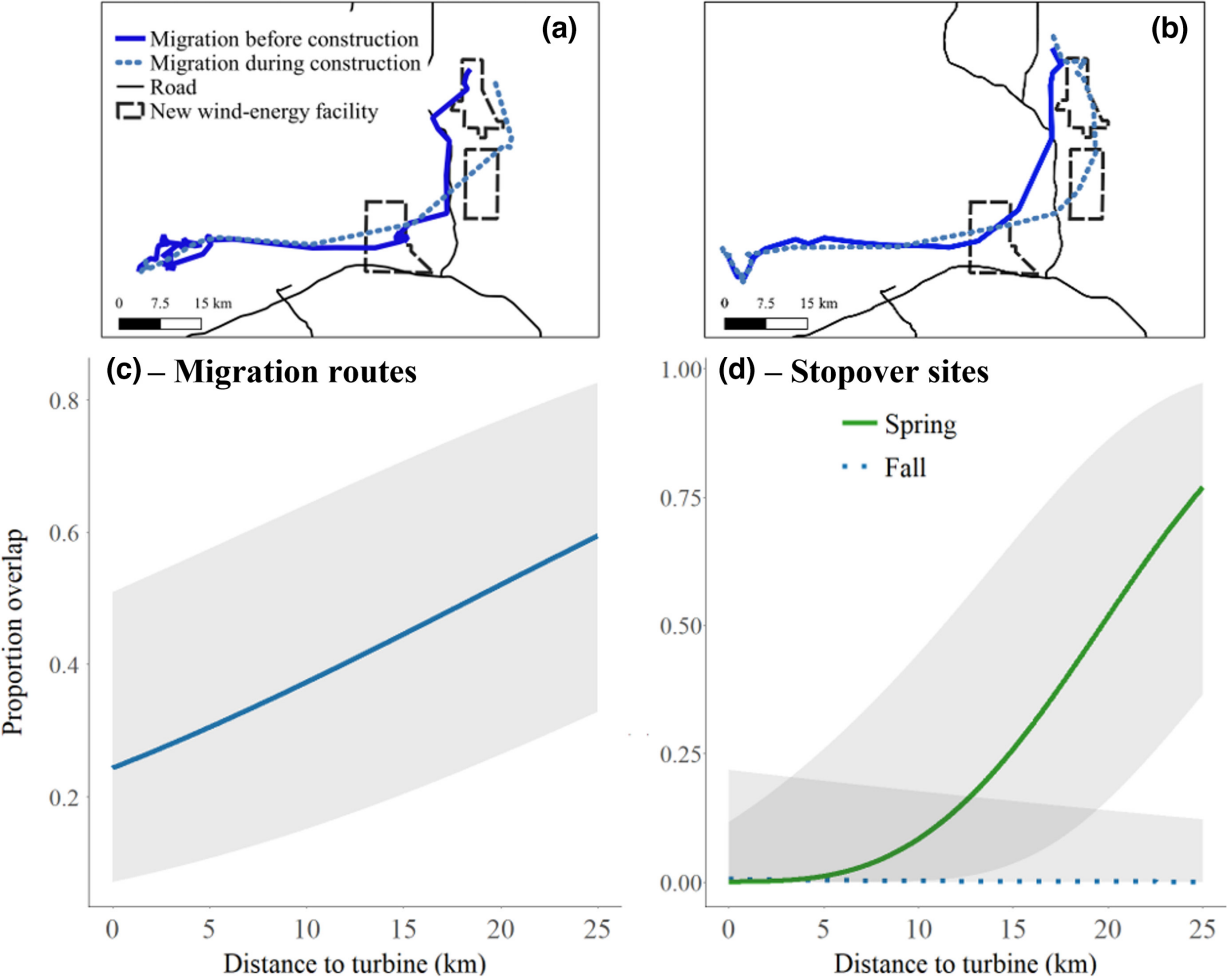
Examples of migrations before and during construction for the same individuals (a and b) and predicted fidelity (proportion overlap ±95% credible intervals) for migration routes (c) and stopover sites (d) of pronghorn in south‐central Wyoming, USA, 2010–2012 and 2018–2020. The proportion overlap was calculated on an individual basis as the proportion of an individual's migration route or stopover from the second monitoring year that overlapped the same individual's route in the first monitoring year.

Average proportion overlap of migration routes for individuals tracked multiple years was 0.29 ± 0.04 (range = 0.01–0.62) in spring and 0.30 ± 0.03 (range = 0.04–0.85) in fall. For stopovers, the average proportion overlap was 0.06 ± 0.03 (range: 0.00 to 0.53) for spring and 0.04 ± 0.01 (range: 0.00 to 0.26) for fall. Fidelity to migration routes increased farther from the new wind‐energy facilities in both seasons (Figure 3, Table S4). Fidelity to stopover sites increased farther from turbines in spring, whereas there was no strong relationship in fall (Figure 3, Table S4).

### 4.3 Route and stopover selection

**FIGURE 4:**
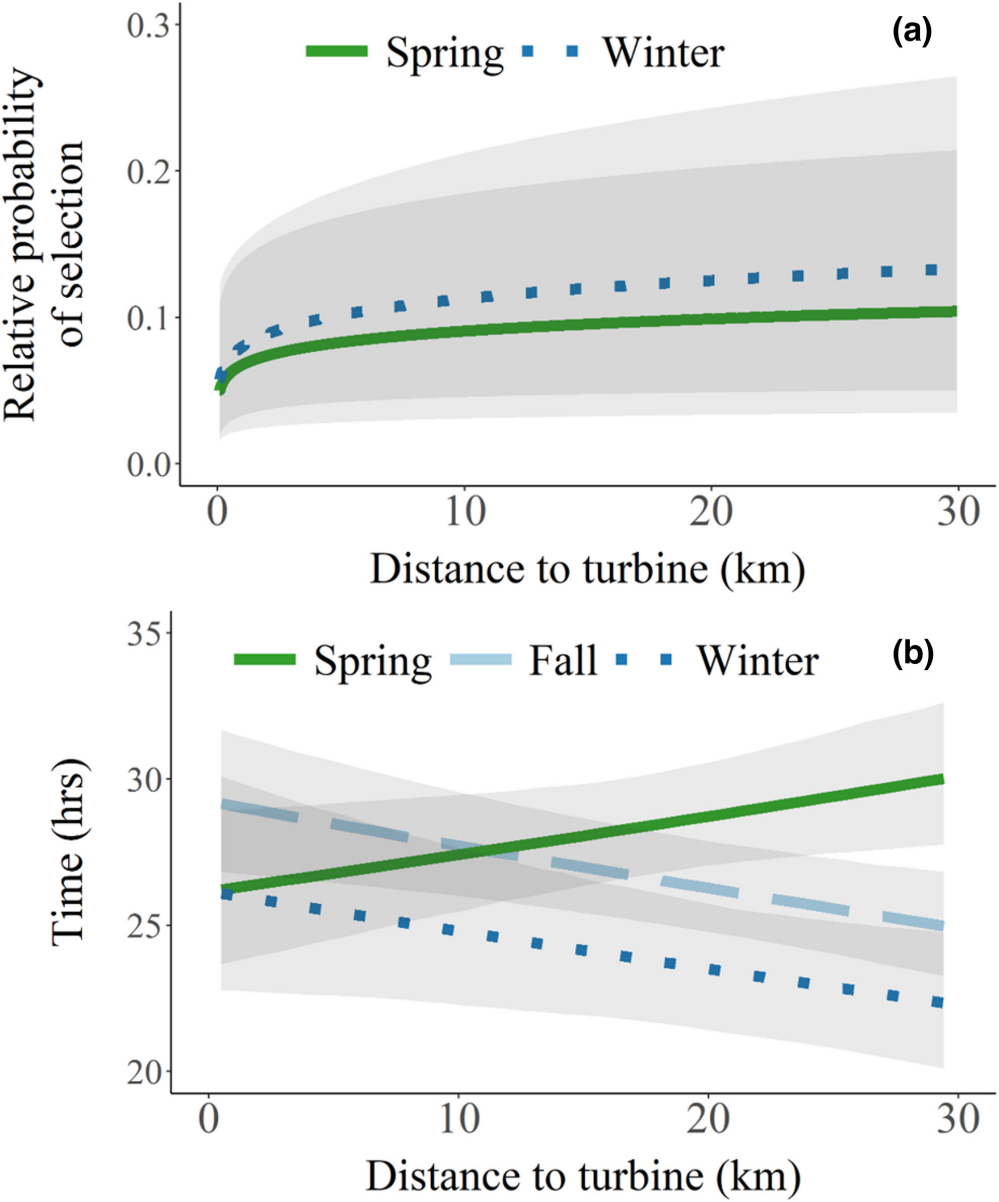
Predicted relative probability of selection (±95% credible intervals) for stopover sites (a) and the predicted amount of time spent in stopover sites relative to distance to turbine (b) for migrating pronghorn relative to turbines in south‐central Wyoming, USA, 2010–2012 and 2018–2020.

For route selection, distance to turbine was only important in fall, when female pronghorn tended to select migration routes closer to turbines than expected, after accounting for habitat variables (Table 1, Table S5). Distance to turbine under construction was only important for route selection in the fall (Table S6), when pronghorn selected migration routes closer to turbines under construction than expected (*β* = −0.77, 95% CIs: −1.24 to −0.29). Pronghorn selected stopover sites farther from turbines in spring and winter, but not fall (Figure 4, Table S5). An increase in distance to turbine from 0 to 1 km translated to a 33% and 300% increase in the relative probability of selection in spring and winter, respectively. Pronghorn also spent more time in stopovers that were farther away from turbines but only during spring migration (Figure 4, Table S7). Distance to turbine under construction was not important for stopover selection in either spring or fall (Table S6).

**TABLE 1:** Coefficient estimates (*β*), 95% credible intervals (CI), and probabilities that pronghorn were selecting for a given variable (*p*(*β* > 0)) for route, stopover site, and small‐scale habitat selection of migrating female pronghorn in south‐central Wyoming, USA, 2010–2012 and 2018–2020

Variable	Spring		Fall		Winter	
	*β* (95% CI)	*p*(*β* > 0)	*β* (95% CI)	*p*(*β* > 0)	*β* (95% CI)	*p*(*β* > 0)
Route selection						
Sagebrush	1.41 (0.72 to 2.14)	1.00	1.42 (0.87 to 2.00)	1.00	−0.56 (−1.74 to 0.63)	.18
Herbaceous	–	–	0.38 (−0.35 to 1.13)	.84	0.44 (−0.86 to 1.85)	.73
TRI	−5.55 (−6.95 to −4.31)	.00	−2.16 (−2.80 to −1.56)	.00	−0.05 (−0.94 to 0.80)	.46
iNDVI	−5.69 (−8.60 to −3.09)	.00	−3.30 (−4.43 to −2.25)	.00	−5.48 (−7.95 to −3.34)	.00
IRG	0.43 (−0.87 to 1.75)	.74	–	–	–	–
Snow depth	–	–	–	–	−1.02 (−3.80 to 2.04)	.24
Dist. to road	–	–	–	–	−1.19 (−2.02 to −0.40)	.00
Dist. to fence	–	–	–	–	−1.07 (−1.84 to −0.37)	.00
Dist. to turbine	–	–	−0.41 (−0.65 to −0.16)	.00	–	–
Stopover selection						
Sagebrush	0.09 (0.003 to 0.18)	.97	0.19 (0.11 to 0.27)	1.00	–	–
Herbaceous	–	–	0.22 (0.11 to 0.34)	1.00	–	–
TRI	−0.47 (−0.61 to −0.34)	.00	–	–	–	–
iNDVI	−0.18 (−0.38 to 0.02)	.04	−0.30 (−0.43 to −0.17)	.00	−0.29 (−0.46 to −0.13)	.00
IRG	−0.10 (−0.70 to 0.51)	.37	–	–	–	–
Dist. to fence	–	–	−0.08 (−0.15 to 0.001)	.03	–	–
Dist. to turbine	0.15 (0.05 to 0.25)	1.00	–	–	0.17 (−0.01 to 0.36)	.97
Step‐selection functions						
Sagebrush	0.02 (−0.08 to 0.11)	.64	0.14 (0.08 to 0.20)	1.00	−0.15 (−0.25 to −0.05)	.00
Herbaceous	0.12 (0.03 to 0.21)	.99	0.08 (0.04 to 0.12)	1.00	0.13 (−0.001 to 0.30)	.97
TRI	−0.22 (−0.32 to −0.14)	.00	−0.20 (−0.25 to −0.14)	.00	−0.16 (−0.29 to −0.03)	.01
iNDVI	−0.19 (−0.30 to −0.08)	.00	−0.10 (−0.15 to −0.05)	.00	−0.28 (−0.44 to −0.14)	.00
IRG	−0.01 (−0.10 to 0.09)	.44	–	–	–	–
Snow depth	–	–	0.06 (−0.36 to 0.49)	.61	–	–
Dist. to road	0.16 (0.08 to 0.25)	1.00	0.03 (−0.02 to 0.08)	.91	0.0002 (−0.12 to 0.13)	.49
Dist. to fence	–	–	−0.04 (−0.08 to −0.003)	.02	–	
Dist. to turbine	0.14 (−0.03 to 0.31)	.95	−0.002 (−0.15 to 0.15)	.49	0.28 (−0.004 to 0.57)	.97
Dist. to turbine:cos(turn angle)	−0.04 (−0.17 to 0.08)	.22	0.0002 (−0.02 to 0.02)	.59	–	
log(step length)	0.03 (0.003 to 0.06)	.96	0.002 (0.02 to 0.02)	.57	0.05 (0.01 to 0.08)	.99
cos(turn angle)	0.05 (−0.04 to 0.14)	.84	−0.05 (−0.09 to −0.005)	.02	−0.08 (−0.18 to 0.02)	.06

Pronghorn selected migration routes that had higher sagebrush cover in the spring and fall and lower herbaceous cover in the spring (Table 1). Pronghorn selected winter migration routes that were closer to both roads and fences than expected (Table 1). Migration routes in all seasons had lower terrain ruggedness and integrated NDVI than expected (Table 1). In the spring, migrating pronghorn selected stopover sites with greater sagebrush cover and lower terrain ruggedness (Table 1). In the fall, pronghorn selected stopover sites with greater sagebrush and herbaceous cover that were closer to fences (Table 1). Pronghorn selected stopover sites with lower integrated NDVI across all seasons (Table 1).

### 4.4 Small‐scale selection

**FIGURE 5:**
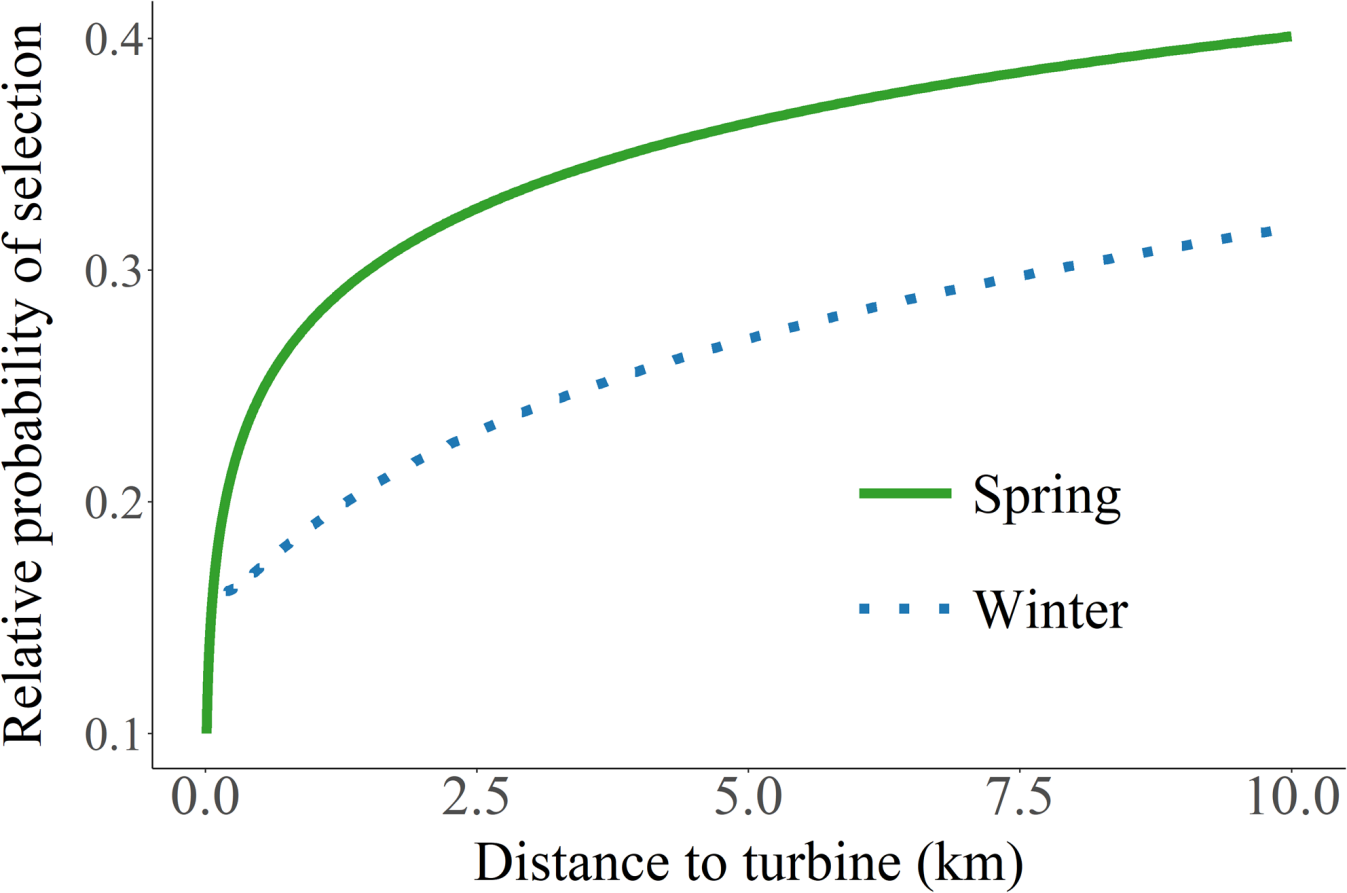
Predicted relative probability of selection in relation to distance to turbine (km) from step‐selection functions evaluating small‐scale movement and selection of migrating female pronghorn in south‐central Wyoming, USA, 2010–2012 and 2018–2020. Credible intervals omitted for clarity (see Appendix S1 for full figure).

Distance to turbine was in the top model for all step‐selection functions, with pronghorn selecting steps that were farther from turbines in both spring and winter, but not in fall, after accounting for habitat variables (Table 1, Table S5). An increase in distance to turbine from 0 to 1 km translated to a 180% and 68% increase in the relative probability of selection in spring and winter, respectively. The top model for all seasons included an effect of distance to turbine, representing an effect on small‐scale avoidance. In spring and winter, there was a high probability (.95–.97) that pronghorn selected habitats farther from turbines, although error estimates were large (Figure 5). For spring, the top model also included an interaction between distance to turbine and cos(turn angle), representing an effect of turbines on small‐scale directional persistence, but the variation of turn angles was minimal, suggesting that the effect of distance to turbine on habitat selection was the more important predictor in spring. For fall, the top model included an interaction between distance to turbine and log(step length), representing an effect of turbines on speed, but credible intervals for both the effect of turbines and the interaction completely overlapped zero.

Pronghorn tended to select for greater sagebrush and herbaceous cover, although there was no evidence of selection for sagebrush in winter (Table 1). Pronghorn also selected for less rugged landscapes and areas with lower integrated NDVI (Table 1). Pronghorn tended to select areas farther from roads in spring and fall and closer to fences in the fall (Table 1).

## 5 DISCUSSION

While pronghorn continued to migrate through wind‐energy facilities, we observed important effects of development on both small‐scale habitat selection and movement behavior during migration. Negative effects were most prominent and consistent during spring migration, which is a critical time for individuals to access important forage resources to support parturition. Pronghorn traveled faster near turbines during spring, with speeds increasing 0.14 km/h for every 5 km closer to wind turbines, although pronghorn traveled slower near turbines during fall migration likely due to turbines being constructed in high‐quality habitat. Wind turbines negatively affected fidelity, with pronghorn migrating near turbines being less faithful to migration routes in both spring and fall and stopover sites in spring. Pronghorn also selected stopover sites farther from turbines in spring and winter and spent less time in stopovers near turbines during spring migration. Finally, pronghorn selected small‐scale steps along the migration route that were farther from turbines than expected in both spring and winter. Overall, our results suggest that wind‐energy development did not pose a barrier to migrating pronghorn, but that pronghorn made important behavioral adjustments relative to turbines, particularly during spring migration, that likely reduced the functional benefits of their seasonal migrations.

We documented a high proportion of pronghorn migrating through wind‐energy facilities (70% within 1 km of wind turbines) and selection of routes closer to turbines during the fall, which may be attributed to the construction of turbines on historical migration routes. Maintenance of such connectivity does not indicate whether the functional benefits of migration remain (Sawyer et al., 2013), stressing the importance of evaluating the effects of development at multiple scales and life‐history stages. We found evidence that pronghorn increased speed near turbines during spring migration, which is consistent with previous studies that found mule deer responded behaviorally to oil and gas development by speeding up near gas wells and is potentially driven by increased noise and human disturbance (Lendrum et al., 2012; Sawyer et al., 2013; Wyckoff et al., 2018). Higher movement rates suggest that wind‐energy facilities increased energy expenditures (Parker et al., 1984) of pronghorn near turbines during spring, with potential effects on foraging opportunities and future fitness (Sawyer et al., 2013; Wyckoff et al., 2018). In contrast to spring migration, pronghorn slowed down near turbines in fall, and we did not find an effect of turbines under construction on speed for individuals migrating in the same area before and during construction, although sample sizes prevented us from evaluating seasons separately. Wind‐energy facilities were constructed in high‐quality pronghorn habitat, which could be ultimately responsible for pronghorn traveling more slowly near turbines in fall, with pronghorn taking advantage of important resources despite the turbines. In addition, snow depth was correlated with distance to turbine under construction and could explain the lack of difference before and during construction if pronghorn slow down due to the difficulty of moving through deep snow. Alternatively, hunting pressure could be affecting pronghorn during fall migration, as has been shown for migrating elk (Mikle et al., 2019), with wind‐energy facilities potentially representing refugia. Regardless, changes in migratory movements and speed can impact access to forage and other functional benefits of migration, which is particularly important during spring migration (Sawyer et al., 2013), and, as a result, development could reduce the ecological benefits of migration (Wyckoff et al., 2018).

Pronghorn also exhibited small‐scale behavioral adjustments, including altering both stopover site and small‐scale habitat selection near turbines. Stopover sites, which are used by migratory ungulates as important foraging and resting habitat (Sawyer et al., 2009; Sawyer & Kauffman, 2011), are critical for maintaining the functional connectivity of a migration route (Sawyer et al., 2013). Our results suggest that pronghorn selected stopover sites farther from turbines in both spring and winter, which could have pushed them into lower quality habitat with reduced foraging success (Sawyer et al., 2013; Wyckoff et al., 2018). Pronghorn also spent less time in stopover sites near turbines during spring migration, which could reduce foraging opportunities near turbines, although we observed the opposite pattern in fall. Reduced use of stopovers near turbines is consistent with previous research suggesting that mule deer continue to migrate through oil and gas development (Lendrum et al., 2012; Wyckoff et al., 2018), but that the intensity of use often declines (Sawyer et al., 2013, 2020) and mule deer shift their stopover sites away from areas of high development (Wyckoff et al., 2018). Altered stopover site selection and small‐scale avoidance can result in functional habitat loss, where the areas around turbines are no longer available to pronghorn (Sawyer et al., 2017), which is especially important during spring migration when ungulates need access to high‐quality forage resources to support reproduction. Demographic consequences of such behavioral modification may have important implications for population dynamics (Sawyer et al., 2017), but future research could quantify the potential survival or reproductive costs when migratory behaviors are disrupted (Runge & Marra, 2005).

Detecting disruptions and effects of development on the migrations of more nomadic or variable species, such as pronghorn (Fryxell et al., 2005; Morrison et al., 2021; Mueller et al., 2011; Sawyer et al., 2013), can be more difficult than for species like mule deer that exhibit high fidelity to narrow pathways (Sawyer et al., 2009; Sawyer & Kauffman, 2011; Wyckoff et al., 2018). Our results suggest that 63% of migratory animals in our study were conditionally migratory, which is consistent with many other studies of pronghorn (Collins, 2016; Jacques et al., 2009; Jakes et al., 2018; Kolar et al., 2011; Larkins et al., 2018; Tack et al., 2019). Nevertheless, when pronghorn did migrate, fidelity to individual routes was similar to that estimated for mule deer in southwestern Wyoming (Wyckoff et al., 2018) and was lower for pronghorn migrating near wind turbines, which highlights the importance of long‐term studies to evaluate the effects of development. Our data were unique in that they allowed us to evaluate migratory behavior prior to the development of two large wind‐energy facilities. Our results suggest pronghorn migrating near turbines under construction were less faithful to their migration routes in both spring and fall and stopover sites in spring compared with pronghorn migrating farther from turbines. The observed lower fidelity could potentially be due to increased disruptions from construction activity causing pronghorn to alter their migration. However, this contrasts with mule deer, whose fidelity to both routes and stopovers was not affected by development, although there was a trend toward reduced fidelity for mule deer in a high‐development area (Wyckoff et al., 2018). Decreased fidelity could eventually lead to the loss of specific migration routes and the associated fitness benefits (Sawyer et al., 2013). Our results suggest that energy development can have important consequences even for variable species that could reduce the persistence of both migratory behavior and populations (Bolger et al., 2008; Harris et al., 2009; Mueller et al., 2011).

Although most studies of pronghorn have simply characterized the presence of migratory behavior (e.g., Jacques et al., 2009), our results are generally consistent with the few that evaluated the effects of habitat factors on selection during migration. Notably, we found no evidence that pronghorn tracked forage green‐up across the landscape, which contrasts with migratory mule deer (Aikens et al., 2017). However, pronghorn often do not migrate along an altitudinal gradient like mule deer, so relatively small changes in forage resources and phenology along the migration routes for pronghorn in our study likely limited the benefits of tracking forage green‐up. Consistent with previous studies of pronghorn both during migration and on seasonal ranges (Seidler et al., 2015; Sheldon & Lindzey, 2005), we found fine‐scale avoidance of roads, which can act as a barrier to movement and represent an additional negative effect of development.

Migration is an important behavioral strategy that is critical for population persistence (Fryxell & Sinclair, 1988; Harris et al., 2009; Sawyer et al., 2009). Ungulate migrations have been increasingly fragmented and disrupted by anthropogenic activities, including energy extraction and the associated development of roads, fences, and pipelines. In our study, pronghorn made behavioral adjustments, including altered speed and stopover site selection during the spring with effects in the fall being more mixed, relative to wind turbines that could impact the functional and fitness benefits of their migration even though connectivity was maintained. We also documented reduced fidelity with the construction of wind‐energy facilities during both spring and fall, which can have implications for the persistence of migration routes. Migration is a unique behavior that is difficult to restore, especially after routes are lost in a population (Jesmer et al., 2018). As development continues to accumulate, behavioral adjustments during migration could lead to population‐level consequences, such as fitness impacts from reduced foraging opportunities or the loss of specific migration routes that may only manifest in the long term. Development thresholds that cause the loss of existing migrations remain unknown for ungulates, but our study clarifies that wind‐energy development is among the anthropogenic factors that can disrupt migratory behavior across formerly intact landscapes.

## AUTHOR CONTRIBUTIONS

**Megan C. Milligan:** Conceptualization (equal); data curation (lead); formal analysis (lead); investigation (lead); methodology (lead); project administration (equal); validation (lead); visualization (lead); writing – original draft (lead). **Aaron N. Johnston:** Conceptualization (equal); funding acquisition (equal); investigation (equal); project administration (equal); writing – review and editing (equal). **Jeffrey L. Beck:** Conceptualization (equal); Data curation (equal); funding acquisition (equal); investigation (equal); project administration (equal); writing – review and editing (equal). **Kaitlyn L. Taylor:** Data curation (equal); investigation (equal); writing – review and editing (equal). **Embere Hall:** Investigation (equal); writing – review and editing (equal). **Lee Knox:** Investigation (equal); writing – review and editing (equal). **Teal Cufaude:** Investigation (equal); writing – review and editing (equal). **Cody Wallace:** Data curation (equal); investigation (equal); writing – review and editing (equal). **Geneva Chong:** Funding acquisition (equal); investigation (equal); writing – review and editing (equal). **Matthew J. Kauffman:** Conceptualization (equal); Data curation (equal); funding acquisition (equal); investigation (equal); project administration (equal); writing – review and editing (equal).

## Supporting information


Appendix S1

## Data Availability

Data are available at https://doi.org/10.5066/P9XDUF9I.
