# Supplementary material for: Wind‐energy development alters pronghorn migration at multiple scales
Source: Ecol Evol. 2023 Jan 10;13(1):e9687. doi: 10.1002/ece3.9687 (PMC9831971; doi:10.1002/ece3.9687)
Supplement: Supplementary file 1 — Appendix S1 [file ECE3-13-e9687-s001.docx]

**APPENDIX**

| Table S1. Summary of migration metrics by year and season for migrating female pronghorn in south-central Wyoming, 2010–2020. The average Euclidean distance (km), total path distance (km), speed over the Euclidean distance (km/hr), speed over the total path distance (km/hr), duration (days), median start and stop dates, and range of migration dates are reported for each season in each year, with the sample sizes (# migration routes) noted parenthetically. The Euclidean distance is the straight-line distance from the start to end point of a migration sequence, whereas the total path distance is the cumulative distance of individual step in a sequence. | | | | | | | | | |
| --- | --- | --- | --- | --- | --- | --- | --- | --- | --- |
| **Year** | **Season** | **Euc. dist. (km)** | **Path dist. (km)** | **Speed (km/hr; Euc. dist.)** | **Speed (km/hr; Path dist.)** | **Duration (days)** | **Start date** | **End date** | **Range of mig. dates** |
| 2010 | spring (8) | 48.6 ± 9.5 | 89.3 ± 23.4 | 0.20 ± 0.05 | 0.28 ± 0.04 | 15.4 ± 5.1 | 11-Apr | 20-Apr | 17 Mar – 26 May |
| 2011 | spring (10) | 44.7 ± 5.0 | 79.1 ± 12.3 | 0.16 ± 0.03 | 0.24 ± 0.03 | 16.9 ± 4.3 | 15-Mar | 31-Mar | 1 Mar – 16 Apr |
| 2012 | spring (7) | 26.2 ± 2.9 | 56.1 ± 7.4 | 0.09 ± 0.02 | 0.6 ± 0.02 | 15.0 ± 2.5 | 5-Mar | 22-Mar | 5 Mar – 13 Apr |
| 2018 | spring (10) | 29.6 ± 3.7 | 61.6 ± 13.0 | 0.18 ± 0.03 | 0.32 ± 0.03 | 9.5 ± 4.0 | 21-Apr | 2-May | 8 Apr – 31 May |
| 2019 | spring (53) | 56.6 ± 3.3 | 95.9 ± 5.1 | 0.21 ± 0.02 | 0.31 ± 0.02 | 14.9 ± 1.3 | 5-Apr | 19-Apr | 11 Mar – 18 May |
| 2020 | spring (33) | 56.2 ± 4.2 | 98.9 ± 6.7 | 0.21 ± 0.04 | 0.30 ± 0.04 | 19.8 ± 2.2 | 23-Feb | 20-Apr | 23 Feb – 18 May |
| 2010 | fall (13) | 54.6± 7.5 | 98.6 ± 15.1 | 0.22 ± 0.03 | 0.33 ± 0.03 | 14.5 ± 2.8 | 16-Nov | 9-Dec | 22 Sept – 28 Dec |
| 2011 | fall (4) | 24.2 ± 8.2 | 85.3 ± 26.3 | 0.06 ± 0.02 | 0.23 ± 0.02 | 14.3 ± 4.7 | 2-Nov | 18-Nov | 2 Nov – 25 Nov |
| 2018 | fall (58) | 47.2 ± 2.6 | 107.9 ± 8.1 | 0.18 ± 0.02 | 0.33 ± 0.02 | 15.4 ± 1.6 | 17-Nov | 29-Nov | 23 Aug – 4 Jan |
| 2019 | fall (48) | 60.3 ± 4.0 | 145.9 ± 13.9 | 0.26 ± 0.03 | 0.43 ± 0.03 | 18.2 ± 2.5 | 27-Oct | 4-Nov | 29 Sept – 31 Dec |
| 2010 | winter (4) | 59.4 ± 17.8 | 95.9 ± 29.3 | 0.19 ± 0.05 | 0.29 ± 0.05 | 17.3 ± 7.5 | 13-Dec | 28-Dec | 4 Dec – 27 Jan |
| 2011 | winter (3) | 20.1 ± 1.8 | 71.3 ± 19.8 | 0.08 ± 0.03 | 0.21 ± 0.03 | 14.7 ± 5.3 | 7-Jan | 25-Jan | 5 Jan – 27 Jan |
| 2018 | winter (29) | 31.6 ± 2.9 | 81.1 ± 7.9 | 0.10 ± 0.01 | 0.24 ± 0.02 | 15.0 ± 1.7 | 27-Jan | 17-Feb | 15 Nov – 25 Mar |
| 2019 | winter (6) | 24.4 ± 6.1 | 85.0 ± 10.5 | 0.08 ± 0.03 | 0.23 ± 0.04 | 17.3 ± 4.9 | 2-Jan | 17-Jan | 27 Nov – 10 Feb |

| Table S2. Support for effects on migration metrics of female pronghorn in south-central Wyoming, 2010–2020. | |
| --- | --- |
| **Model** | **WAIC** |
| **Euclidean distance** |  |
| Season | 6579.88 |
| Prop. route within 1 km of turbines × season | 6582.36 |
| Null | 6598.96 |
| Prop. route within 1 km of turbines | 6599.92 |
| **Total distance** |  |
| Season | 7113.14 |
| Prop. route within 1 km of turbines × season | 7116.47 |
| Null | 7131.08 |
| Prop. route within 1 km of turbines | 7132.77 |
| **Speed (Euclidean distance)** |  |
| Season | -259.66 |
| Prop. route within 1 km of turbines × season | -258.06 |
| Prop. route within 1 km of turbines | -251.06 |
| Null | -248.65 |
| **Speed (Total distance)** |  |
| Season | -253.78 |
| Prop. route within 1 km of turbines × season | -251.23 |
| Prop. route within 1 km of turbines | -233.56 |
| Null | -230.62 |
| **Duration** |  |
| Null | 2250.47 |
| Prop. route within 1 km of turbines | 2251.2 |
| Season | 2254.32 |
| Prop. route within 1 km of turbines × season | 2257.72 |

| Table S3. Model selection for speed relative to both habitat and turbine variables for migrating female pronghorn in south-central Wyoming, 2010–2020. | |
| --- | --- |
| **Model** | **WAIC** |
| **Spring** |  |
| IRG + distance to turbine | 17226.60 |
| IRG | 17227.26 |
| IRG × distance to turbine | 17227.44 |
| Distance to turbine | 17242.09 |
| Null | 17243.44 |
| **Fall** |  |
| Sagebrush + distance to turbine | 32209.95 |
| Sagebrush | 32211.46 |
| Sagebrush × distance to turbine | 32213.04 |
| Distance to turbine | 32264.15 |
| Null | 32266.82 |

| Table S4. Model selection for route and stopover fidelity relative to turbines for migrating female pronghorn in south-central Wyoming, 2010–2020. | |
| --- | --- |
| **Model** | **WAIC** |
| **Route Selection** |  |
| Distance to turbine | -44.68 |
| Distance to turbine × season | -42.03 |
| Null | -39.13 |
| Season | -37.40 |
| **Stopover Selection** |  |
| Distance to turbine × season | -565.42 |
| Season | -560.83 |
| Null | -560.82 |
| Distance to turbine | -558.42 |

| Table S5. Support for final candidate models evaluating migration route selection, stopover site selection, and small-scale habitat selection and movement behavior of migrating female pronghorn in south-central Wyoming, 2010-2020, in each season. | |
| --- | --- |
| **Model** | **WAIC** |
| **Route Selection** |  |
| Spring |  |
| Sagebrush + TRI + iNDVI + IRG | 782.58 |
| Sagebrush + TRI + iNDVI + IRG + dist. to turbine | 783.73 |
| Dist. to turbine | 1063.10 |
| Null | 1086.97 |
| Fall |  |
| Sagebrush + herbaceous + TRI + iNDVI + dist. to turbine | 889.24 |
| Sagebrush + herbaceous + TRI + iNDVI | 896.85 |
| Dist. to turbine | 1078.98 |
| Null | 1104.94 |
| Winter |  |
| Sagebrush + herbaceous + TRI + iNDVI + snow depth + dist. to road + dist. to fence | 320.55 |
| Sagebrush + herbaceous + TRI + iNDVI + snow depth + dist. to road + dist. to fence + dist. to turbine | 322.82 |
| Null | 377.30 |
| Dist. to turbine | 377.66 |
| **Stopover Selection** |  |
| **Spring** |  |
| Sagebrush + TRI + iNDVI + IRG + dist. to turbine | 4463.31 |
| Sagebrush + TRI + iNDVI + IRG | 4470.50 |
| Dist. to turbine | 4526.50 |
| Null | 4533.26 |
| **Fall** |  |
| Sagebrush + herbaceous + iNDVI + dist. to fence | 5304.06 |
| Sagebrush + herbaceous + iNDVI + dist. to fence + dist. to turbine | 5305.81 |
| Null | 5353.11 |
| Dist. to turbine | 5354.93 |
| **Winter** |  |
| iNDVI + dist. to turbine | 1964.51 |
| iNDVI | 1966.36 |
| Dist. to turbine | 1976.51 |
| Null | 1984.00 |
| **Step-Selection Functions** |  |
| **Spring** |  |
| Sagebrush + herbaceous + TRI + iNDVI + IRG + dist. to road + dist. to turbine:cos(turn angle) | 12324.76 |
| Sagebrush + herbaceous + TRI + iNDVI + IRG + dist. to road + dist. to turbine:log(step length) | 12336.11 |
| Sagebrush + herbaceous + TRI + iNDVI + IRG + dist. to road + dist. to turbine | 12337.84 |
| Sagebrush + herbaceous + TRI + iNDVI + IRG + dist. to road | 12338.86 |
| Dist. to turbine:cos(turn angle) | 12440.72 |
| Null | 12447.10 |
| Dist. to turbine | 12448.17 |
| Dist. to turbine:log(step length) | 12449.26 |
| **Fall** |  |
| Sagebrush + herbaceous + TRI + iNDVI + snow depth + dist. to road + dist. to fence + dist. to turbine:log(step length) | 37525.25 |
| Sagebrush + herbaceous + TRI + iNDVI + snow depth + dist. to road + dist. to fence + dist. to turbine:cos(turn angle) | 37532.21 |
| Sagebrush + herbaceous + TRI + iNDVI + snow depth + dist. to road + dist. to fence | 37534.65 |
| Sagebrush + herbaceous + TRI + iNDVI + snow depth + dist. to road + dist. to fence + dist. to turbine | 37534.71 |
| Dist. to turbine:cos(turn angle) | 37858.99 |
| Dist. to turbine:log(step length) | 37860.30 |
| Null | 37860.31 |
| Dist. to turbine | 37861.50 |
| **Winter** |  |
| Sagebrush + herbaceous + TRI + iNDVI + dist. to road + dist. to turbine | 7803.86 |
| Sagebrush + herbaceous + TRI + iNDVI + dist. to road + dist. to turbine:log(step length) | 7805.15 |
| Sagebrush + herbaceous + TRI + iNDVI + dist. to road + dist. to turbine:cos(turn angle) | 7805.35 |
| Sagebrush + herbaceous + TRI + iNDVI + dist. to road | 7808.50 |
| Dist. to turbine | 7882.63 |
| Dist. to turbine:log(step length) | 7884.44 |
| Dist. to turbine:cos(turn angle) | 7884.46 |
| Null | 7885.24 |

| Table S6. Support for final candidate models evaluating migration route and stopover site selection of migrating female pronghorn in south-central Wyoming, 2010–2020, in each season during which construction occurred. Construction occurred in spring 2010 and 2020 and fall 2019. | |
| --- | --- |
| **Model** | **WAIC** |
| **Route Selection** |  |
| **Spring** |  |
| TRI | 282.08 |
| TRI + dist. to turbine | 284.32 |
| Dist. to turbine | 351.37 |
| Null | 359.29 |
| **Fall** |  |
| Sagebrush + TRI + iNDVI + dist. to turbine | 328.66 |
| Sagebrush + TRI + iNDVI | 335.30 |
| Dist. to turbine | 418.40 |
| Null | 431.19 |
| **Stopover Selection** |  |
| **Spring** |  |
| TRI + iNDVI | 1150.08 |
| Null | 1150.79 |
| TRI + iNDVI + dist. to turbine | 1151.92 |
| Dist. to turbine | 1152.87 |
| **Fall** |  |
| Sagebrush + TRI + iNDVI | 2325.74 |
| Sagebrush + TRI + iNDVI + dist. to turbine | 2326.17 |
| Dist. to turbine | 2334.41 |
| Null | 2336.18 |

| Table S7. Support for effects of variables influencing the time spent in stopover sites by migrating female pronghorn in south-central Wyoming, 2010–2020. | |
| --- | --- |
| Model | WAIC |
| Distance to turbine × season | 4118.75 |
| Distance to turbine | 4120.12 |
| Null | 4121.10 |
| Season | 4123.06 |

| Table S8. Support for effects of habitat variables on route selection of migrating female pronghorn in south-central Wyoming, 2010–2020, in each season. | |
| --- | --- |
| **Model** | **WAIC** |
| **Spring** |  |
| TRI | 815.56 |
| iNDVI | 993.05 |
| Sagebrush | 1060.67 |
| IRG | 1082.29 |
| Null | 1086.97 |
| Herbaceous | 1088.29 |
| **Fall** |  |
| TRI | 966.42 |
| iNDVI | 1022.83 |
| Sagebrush | 1083.80 |
| Herbaceous | 1104.70 |
| Null | 1104.94 |
| Snow depth | 1107.63 |
| **Winter** |  |
| iNDVI | 325.33 |
| Herbaceous | 355.89 |
| TRI | 368.60 |
| Snow depth | 370.53 |
| Sagebrush | 372.67 |
| Null | 377.30 |

| Table S9. Support for effects of anthropogenic variables on route selection of migrating female pronghorn in south-central Wyoming, 2010–2020, in each season. | |
| --- | --- |
| **Model** | **WAIC** |
| **Spring** |  |
| Sagebrush + TRI + iNDVI + IRG | 782.58 |
| Sagebrush +TRI + iNDVI + IRG + dist. to road | 783.86 |
| Sagebrush + TRI + iNDVI + IRG + dist. to fence | 784.79 |
| Sagebrush +TRI + iNDVI + IRG + dist. to road + dist. to fence | 786.11 |
| Null | 1086.97 |
| Dist. to fence | 1087.56 |
| Dist. to road | 1087.57 |
| Dist. to road + dist. to fence | 1087.89 |
| **Fall** |  |
| Sagebrush + herbaceous + TRI + iNDVI | 896.85 |
| Sagebrush + herbaceous + TRI + iNDVI + dist. to fence | 898.10 |
| Sagebrush + herbaceous + TRI + iNDVI + dist. to road | 898.40 |
| Sagebrush + herbaceous + TRI + iNDVI + dist. to road + dist. to fence | 899.81 |
| Dist. to road | 1101.84 |
| Dist. to road + dist. to fence | 1103.64 |
| Null | 1104.94 |
| Dist. to fence | 1106.81 |
| **Winter** |  |
| Sagebrush + herbaceous + TRI + iNDVI + snow depth + dist. to road + dist. to fence | 320.55 |
| Sagebrush + herbaceous + TRI + iNDVI + snow depth + dist. to road | 326.88 |
| Sagebrush + herbaceous + TRI + iNDVI + snow depth | 335.29 |
| Dist. to fence | 368.91 |
| Dist. to road + dist. to fence | 369.05 |
| Dist. to road | 375.72 |
| Null | 377.30 |
| Sagebrush + herbaceous + TRI + iNDVI + snow depth + dist. to fence | 466.53 |

| Table S10. Support for effects of habitat variables on stopover site selection of migrating female pronghorn in south-central Wyoming, 2010–2020, in each season. | |
| --- | --- |
| **Model** | **WAIC** |
| **Spring** |  |
| TRI | 4474.51 |
| iNDVI | 4524.10 |
| IRG | 4532.09 |
| Sagebrush | 4533.18 |
| Null | 4533.26 |
| Herbaceous | 4534.53 |
| **Fall** |  |
| Sagebrush | 5325.26 |
| iNDVI | 5348.19 |
| Herbaceous | 5348.70 |
| Null | 5353.11 |
| Snow depth | 6000.81 |
| TRI | 7503.20 |
| **Winter** |  |
| iNDVI | 1966.36 |
| Null | 1984.00 |
| Herbaceous | 1984.74 |
| Snow depth | 1984.75 |
| TRI | 1985.19 |
| Sagebrush | 1985.40 |

| Table S11. Support for effects of anthropogenic variables on stopover site selection of migrating female pronghorn in south-central Wyoming, 2010–2020, in each season. | |
| --- | --- |
| **Model** | **WAIC** |
| **Spring** |  |
| Sagebrush + TRI + iNDVI + IRG | 4470.50 |
| Sagebrush +TRI + iNDVI + IRG + dist. to fence | 4470.93 |
| Sagebrush + TRI + iNDVI + IRG + dist. to road | 4471.92 |
| Sagebrush +TRI + iNDVI + IRG + dist. to road + dist. to fence | 4472.40 |
| Null | 4533.26 |
| Dist. to fence | 4534.22 |
| Dist. to road | 4534.77 |
| Dist. to road + dist. to fence | 4535.76 |
| **Fall** |  |
| Sagebrush + herbaceous + iNDVI + dist. to fence | 5304.06 |
| Sagebrush + herbaceous + iNDVI + dist. to road + dist. to fence | 5305.39 |
| Sagebrush + herbaceous + iNDVI | 5305.89 |
| Sagebrush + herbaceous + iNDVI + dist. to road | 5307.40 |
| Null | 5353.11 |
| Dist. to fence | 5353.28 |
| Dist. to road | 5354.78 |
| Dist. to road + dist. to fence | 5354.87 |
| **Winter** |  |
| iNDVI | 1966.36 |
| iNDVI + dist. to fence | 1967.86 |
| iNDVI + dist. to road | 1967.97 |
| iNDVI + dist. to road + dist. to fence | 1969.54 |
| Null | 1984.00 |
| Dist. to road | 1984.94 |
| Dist. to fence | 1985.58 |
| Dist. to road + dist. to fence | 1986.60 |

| Table S12. Support for effects of habitat variables on small-scale habitat selection and movement behavior of migrating female pronghorn in south-central Wyoming, 2010-2020, in each season. | |
| --- | --- |
| **Model** | **WAIC** |
| **Spring** |  |
| TRI | 12408.87 |
| iNDVI | 12426.22 |
| Sagebrush | 12426.76 |
| Herbaceous | 12427.33 |
| IRG | 12445.65 |
| Null | 12447.10 |
| **Fall** |  |
| TRI | 37735.52 |
| Sagebrush | 37761.57 |
| Herbaceous | 37816.57 |
| Snow depth | 37834.33 |
| iNDVI | 37845.07 |
| Null | 37860.31 |
| **Winter** |  |
| iNDVI | 7855.69 |
| TRI | 7862.75 |
| Sagebrush | 7874.39 |
| Herbaceous | 7876.65 |
| Null | 7885.24 |
| Snow depth | 7886.47 |

| Table S13. Support for effects of anthropogenic variables on small-scale habitat selection and movement behavior of migrating female pronghorn in south-central Wyoming, 2010-2020, in each season. | |
| --- | --- |
| **Model** | **WAIC** |
| **Spring** |  |
| Sagebrush + herbaceous + TRI + iNDVI + IRG + dist. to road + dist. to fence | 12338.11 |
| Sagebrush + herbaceous + TRI + iNDVI + IRG + dist. to road | 12338.86 |
| Sagebrush + herbaceous + TRI + iNDVI + IRG | 12353.12 |
| Sagebrush + herbaceous + TRI + iNDVI + IRG + dist. to fence | 12353.26 |
| Dist. to road | 12436.08 |
| Dist. to fence | 12446.91 |
| Dist. to road + dist. to fence | 12446.91 |
| Null | 12447.10 |
| **Fall** |  |
| Sagebrush + herbaceous + TRI + iNDVI + snow depth + dist. to road + dist. to fence | 37534.65 |
| Sagebrush + herbaceous + TRI + iNDVI + snow depth + dist. to road | 37537.59 |
| Sagebrush + herbaceous + TRI + iNDVI + snow depth + dist. to fence | 37563.11 |
| Sagebrush + herbaceous + TRI + iNDVI + snow depth | 37570.20 |
| Dist. to road | 37831.54 |
| Dist. to fence | 37851.29 |
| Dist. to road + dist. to fence | 37851.40 |
| Null | 37860.31 |
| **Winter** |  |
| Sagebrush + herbaceous + TRI + iNDVI + dist. to road | 7808.50 |
| Sagebrush + herbaceous + TRI + iNDVI + dist. to road + dist. to fence | 7809.07 |
| Sagebrush + herbaceous + TRI + iNDVI | 7814.19 |
| brush + herbaceous + TRI + iNDVI + dist. to fence | 7815.61 |
| Dist. to road | 7880.00 |
| Dist. to road + dist. to fence | 7884.85 |
| Dist. to fence | 7884.85 |
| Null | 7885.24 |

| Table S14. Support for effects of habitat variables on migration route selection of migrating female pronghorn in south-central Wyoming, 2010–2020, in each season during which construction was occurring. | |
| --- | --- |
| **Model** | **WAIC** |
| **Spring** |  |
| TRI | 282.08 |
| Sagebrush | 347.90 |
| Null | 359.29 |
| iNDVI | 360.17 |
| IRG | 360.79 |
| Herbaceous | 361.48 |
| **Fall** |  |
| iNDVI | 384.171 |
| TRI | 408.619 |
| Sagebrush | 408.803 |
| Snow depth | 429.547 |
| Herbaceous | 430.532 |
| Null | 431.185 |

| Table S15. Support for effects of anthropogenic variables on migration route selection of migrating female pronghorn in south-central Wyoming, 2010–2020, in each season during which construction was occurring. | |
| --- | --- |
| **Model** | **WAIC** |
| **Spring** |  |
| TRI | 282.08 |
| TRI + dist. to road | 283.95 |
| TRI + dist. to fence | 285.03 |
| TRI + dist. to road + dist. to fence | 287.03 |
| Null | 359.29 |
| Dist. to road | 360.90 |
| Dist. to fence | 362.23 |
| Dist. to road + dist. to fence | 363.72 |
| **Fall** |  |
| Sagebrush + TRI + iNDVI | 335.30 |
| Sagebrush + TRI + iNDVI + dist. to road | 335.74 |
| Sagebrush + TRI + iNDVI + dist. to fence | 336.54 |
| Sagebrush + TRI + iNDVI + dist. to road + dist. to fence | 338.07 |
| Dist. to road + dist. to fence | 426.79 |
| Dist. to fence | 426.95 |
| Dist. to road | 430.31 |
| Null | 431.19 |

| Table S16. Support for effects of habitat variables on stopover site selection of migrating female pronghorn in south-central Wyoming, 2010–2020, in each season during which construction was occurring. | |
| --- | --- |
| **Model** | **WAIC** |
| **Spring** |  |
| TRI | 1149.86 |
| iNDVI | 1149.964 |
| Null | 1150.793 |
| Sagebrush | 1151.926 |
| Herbaceous | 1152.313 |
| IRG | 1152.464 |
| **Fall** |  |
| Sagebrush | 2329.459 |
| iNDVI | 2334.424 |
| TRI | 2335.63 |
| Null | 2336.181 |
| Herbaceous | 2336.884 |
| Snow depth | 2337.18 |

| Table S17. Support for effects of anthropogenic variables on stopover site selection of migrating female pronghorn in south-central Wyoming, 2010–2020, in each season during which construction was occurring. | |
| --- | --- |
| **Model** | **WAIC** |
| **Spring** |  |
| TRI | 1150.08 |
| TRI + dist. to fence | 1150.45 |
| Null | 1150.79 |
| TRI + dist. to road | 1150.86 |
| TRI + dist. to road + dist. to fence | 1151.33 |
| Dist. to fence | 1151.42 |
| Dist. to road | 1152.04 |
| Dist. to road + dist. to fence | 1152.73 |
| **Fall** |  |
| Sagebrush + TRI + iNDVI | 2325.74 |
| Sagebrush + TRI + iNDVI + dist. to road | 2326.95 |
| Sagebrush + TRI + iNDVI + dist. to road + dist. to fence | 2327.46 |
| Null | 2336.18 |
| Dist. to road | 2337.10 |
| Dist. to fence | 2337.23 |
| Dist. to road + dist. to fence | 2338.08 |
| Sagebrush + TRI + iNDVI + dist. to fence | 2364.67 |


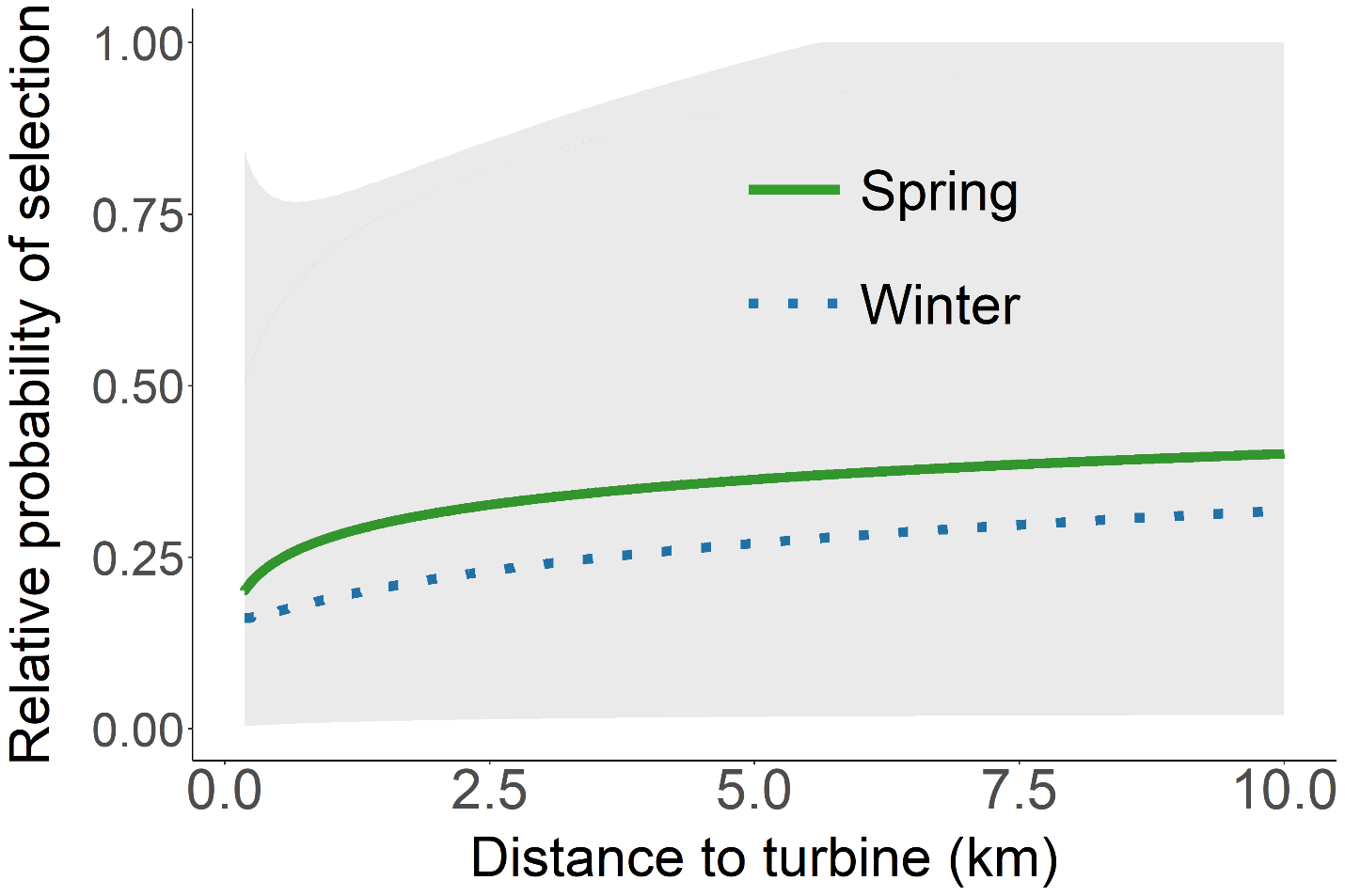


Figure S1. Predicted relative probability of selection in relation to distance to turbine (km) from step-selection functions evaluating small-scale movement and selection of migrating female pronghorn in south-central Wyoming, USA, 2010–2012 and 2018–2020.
